# Apoptosis and the activity of ceramide, Bax and Bcl-2 in the lungs of neonatal rats exposed to limited and prolonged hyperoxia

**DOI:** 10.1186/1465-9921-7-100

**Published:** 2006-07-26

**Authors:** Ahmad W Husari, Ghassan S Dbaibo, Hala Bitar, Aline Khayat, Shoghag Panjarian, Michel Nasser, Fadi F Bitar, Marwan El-Sabban, Ghazi Zaatari, Salman M Mroueh

**Affiliations:** 1 Division of Pulmonary and Critical Care Medicine, Department of Internal Medicine, American University of Beirut-Medical Center, P.O. Box: 113-6044, Beirut 1107 2802, Lebanon; 2 Department of Pediatrics, American University of Beirut-Medical Center, P.O. Box: 113-6044, Beirut 1107 2802, Lebanon; 3 Department of Biochemistry, American University of Beirut-Medical Center, P.O. Box: 113-6044, Beirut 1107 2802, Lebanon; 4 Department of Physiology, American University of Beirut-Medical Center, P.O. Box: 113-6044, Beirut 1107 2802, Lebanon; 5 Department of Human Morphology, American University of Beirut-Medical Center, P.O. Box: 113-6044, Beirut 1107 2802, Lebanon; 6 Pathology Department, American University of Beirut-Medical Center, P.O. Box: 113-6044, Beirut 1107 2802, Lebanon

## Abstract

**Background:**

The aim of the study is to examine the effect of limited and prolonged hyperoxia on neonatal rat lung. This is done by examining the morphologic changes of apoptosis, the expression of ceramide, an important mediator of apoptosis, the expression of inflammatory mediators represented by IL-1β and the expression of 2 proto-oncogenes that appear to modulate apoptosis (Bax and Bcl-2).

**Methods:**

Newborn rats were placed in chambers containing room air or oxygen above 90% for 7 days. The rats were sacrificed at 3, 7 or 14 days and their lungs removed. Sections were fixed, subjected to TUNEL, Hoechst, and E-Cadherin Staining. Sections were also incubated with anti-Bcl-2 and anti-Bax antisera. Bcl-2 and Bax were quantitated by immunohistochemistry. Lipids were extracted, and ceramide measured through a modified diacylglycerol kinase assay. RT-PCR was utilized to assess IL-1β expression.

**Results:**

TUNEL staining showed significant apoptosis in the hyperoxia-exposed lungs at 3 days only. Co-staining of the apoptotic cells with Hoechst, and E-Cadherin indicated that apoptotic cells were mainly epithelial cells. The expression of Bax and ceramide was significantly higher in the hyperoxia-exposed lungs at 3 and 14 days of age, but not at 7 days. Bcl-2 was significantly elevated in the hyperoxia-exposed lungs at 3 and 14 days. IL-1β expression was significantly increased at 14 days.

**Conclusion:**

Exposure of neonatal rat lung to hyperoxia results in early apoptosis documented by TUNEL assay. The early rise in Bax and ceramide appears to overcome the anti-apoptotic activity of Bcl-2. Further exposure did not result in late apoptotic changes. This suggests that apoptotic response to hyperoxia is time sensitive. Prolonged hyperoxia results in acute lung injury and the shifting balance of ceramide, Bax and Bcl-2 may be related to the evolution of the inflammatory process.

## Background

Oxygen supplement is used in different therapeutic interventions. Respiratory diseases in neonates such as hyaline membrane disease and persistent pulmonary hypertension often require high concentrations of supplemental oxygen. Such concentrations are known to cause cellular damage and death in lungs and other organs as well. Cell death secondary to hyperoxia occurs via two mechanisms: cell necrosis and apoptosis [1,2].

Cell necrosis is a mode of cell death that occurs exclusively in environmental disruption under nonphysiological conditions, resulting in inflammatory reactions caused by cell lysis and release of intracellular contents in the extracellular space [3]. Necrosis is always pathological.

Apoptosis or programmed cell death is a gene-regulated process in which a coordinated series of morphological changes such as nucleus and chromatin condensation, cell membrane blebbing and fragmentation of the cell into membrane-bound apoptotic bodies occurs, resulting in cell death [4]. The apoptotic process appears to be regulated by a number of substances that include the sphingolipid ceramide, a sphingolipid breakdown product [5,6] and members of the Bcl-2 family of proteins. Bcl-2 family members determine cell death and survival by controlling mitochondrial membrane ion permeability, cytochrome c release, and the subsequent activation of caspase (caspase 3, caspase 9) executor functions [7]. The Bcl-2 family of proteins consists of pro-apoptotic (Bax, Bcl-X_s_, Bak and Bad) and anti-apoptotic (Bcl-2, Bcl-X_L _and Bcl-w) proteins [8]. The role of Bcl-2 family members in response to hypoxia has been studied in several models. Jonas et al [9] found that minutes of hypoxia trigger the opening of large multi-conductance channels of the outer mitochondrial membranes inside the presynaptic terminal of a squid giant synapse. ZVAD, wide-spectrum caspase inhibitor, inhibits proteolysis of BCL-xL during hypoxia and prevents the appearance of the hypoxia-induced channels. Similarly, Yamamoto et al [10] demonstrated the pro-apoptotic mechanisms of hypoxia in epithelial cells by noticing a significant decrease in Bcl-2 and Bcl-xL, which protects cells from apoptosis. Bax, which promotes apoptosis, was translocated from the cytoplasm to the mitochondrial membrane in response to hypoxia. While there is interest as to the function of the Bcl-2 family members in hypoxia-induced cell death, there are few studies focusing on the effects of hyperoxia, either by itself or together with hypoxia.

The sphingolipid ceramide is an important second signal molecule that regulates diverse signaling pathways involving cell growth and differentiation, gene transcription, viral replication, ligand binding, and cell death [11]. For the most part, ceramide's effects are antagonistic to growth and survival. Ceramide has been shown to play a regulatory role in apoptosis induced by cytokines, DNA injury, and other forms of cell stress [11-13]. In response to exposure to stress, cells activate pathways that result in the accumulation of cellular ceramide. Depending on the cell type and the level of injury, the accumulated ceramide can either induce cell cycle arrest by activation of the retinoblastoma protein, or induce apoptosis by a Bcl-2-regulated mechanism [14-16]. The effects of hyperoxia on ceramide levels have not been studied although hyperoxia decreases sphingomyelin levels in surfactant without affecting the activity of serine palmitoyl transferase, the first enzyme in de novo ceramide synthesis [17]. In this study, we determine the effects of exposure of rat neonates to hyperoxia for a period of one week on the levels of ceramide and the correlation with apoptosis and expression of Bcl-2 and Bax.

## Methodology

### Experimental design

Between 2 and 4 hours following birth, rat pups were distributed to litters of ten of equal body weight and nested on softwood shavings in plexiglass exposure chambers. They were assigned to receive either high oxygen concentration or room air. Food and water were made available ad libitum, and lighting provided on a 12-hr light/dark cycle. Room and chamber temperature were maintained at 22–24°C. Animals received care according to "the Guide for the Care and use of Laboratory Animals of the National Academy of Science and the Principles of Laboratory Animal Care of the National Society of Medical Research". The chambers were equipped with a flow-through system for controlling the delivery of either medical oxygen or room air. Six liters per minute of medical oxygen were flowed through the hyperoxic exposure chambers and oxygen concentration was monitored continuously using an oxygen monitor (Ohmeda 5120, Douisville, CO) to be maintained at 95 ± 2%. In the control chambers, the oxygen concentration was maintained at 21 ± 2%. Carbon dioxide concentrations were maintained at 0.3%, and relative humidity between 50 and 80%. Mothers were switched daily between hyperoxic and room air chambers to prevent maternal oxygen toxicity. At the end of postnatal day 7, hyperoxia-exposed pups were weaned to room air by lowering the oxygen concentration to 60% for 4 hours, to 40% for 4 hours and then to room air. At birth and on postnatal days 3, 7, and 14, all pups were weighed and two from each litter were arbitrarily selected, anesthetized with pentobarbital (50 mg/kg) and their lungs excised.

### Tissue processing

The lungs were excised. The two left lobes were processed and fixed in 4% buffered formalin. After 48 hours of fixation in formalin, the left lobes were paraffin embedded. The right lobes were divided into three specimens. The first specimen was fixed in methanol and was homogenized (Brinkmann Homogenizer model PT 10/35, Switzerland). 400 μl of the homogenate was measured and 2 ml methanol, 1 ml chloroform was added and saved at -80°C. The second specimen was homogenized and stored in Sucrose-HEPES-Tris Cl (SHT) buffer. The second specimen was utilized for Ceramidase and sphingomylinase assays. The third specimen was frozen in liquid nitrogen and utilized later for RT-PCR for expression of IL-1β.

### 1. Morphological changes of apoptosis

#### A. Confocal microscopy

To correlate chromatin alteration with the presence or absence of dUTP labeling, histologic sections were analyzed by fluorescence microscopy. Sections were fixed, subjected to FITC-conjugated TUNEL (Boehringer Mannheim, Germany) assay and then scanned for signal using 488 nm excitation line from the Zeiss LSM 410 laser scanning confocal microscope. Images were reconstructed and examined for apoptosis.

#### B. TUNEL assay

The terminal deoxynucleotidyl transferase-mediated dUTP nick-end labeling (TUNEL) assay was used to monitor the extent of DNA fragmentation as a measure of apoptosis in paraffin-embedded sections. The assay was performed according to the recommendations of the manufacturer (Boehringer Mannheim, Germany). Using paraffin sections that were 3 μm thick, the TUNEL assay was performed as described previously [18]. Fluorescein-conjugated dUTP incorporated in nucleotide polymers was detected and quantified using fluorescence microscopy (Zeiss LSM 410, Germany). Positive and negative controls were used to verify the specificity of the TUNEL assay. Positive controls were treated with DNase I (Sigma Chemical Co.) to enzymatically induce DNA fragmentation. TUNEL-positive nuclei were distinguished from the TUNEL-negative nuclei by counter staining with Hoechst stain and were counted after being photographed.

Lung sections were incubated and stained and the nuclei were visualized. Tissues were then counterstained with Hoechst (Molecular Probes, Eugene, OR). The number of TUNEL positive cells was counted versus the total number of cells labeled by the Hoechst stain. The apoptotic index was calculated by dividing the number of TUNEL positive to the total number of cells. An apoptotic score from 0 to 4 was also derived whereby 0 indicates 0% TUNEL positive cells, 1 indicates 1–25%, 2 indicates 25–50%, 3 indicates 50–75%, and 4 indicates 75–100%.

### 2. Determination of ceramide levels

#### A. Lipid extraction

Lipids were extracted by the method of Bligh and Dyer [19]. Briefly, cell membranes were extracted with 3 ml of chloroform/methanol (1/2). The monophase was mixed, then water was added and the samples rested for 10 minutes. The organic and aqueous phases were subsequently separated by addition of 1 ml of chloroform and 1 ml of water followed by vigorous shaking and centrifugation at 200×. The organic phase was carefully removed and transferred to a new tube and the sample dried under nitrogen. Lipids were then resuspended in 1 ml of chloroform.

#### B. Ceramide measurement

Ceramide levels were measured using a modified diacylglycerol kinase assay using external standards [20]. In this assay, 80% of the lipid sample was dried under nitrogen. The dried lipid was solubilized in octyl-β-D-glucoside/dioleoyl phosphatidylglycerol micellar solution (7.5% octyl-β-D-glucoside, 25 mM dioleoyl phosphatidylglycerol) by 2 cycles of sonication in a bath sonicator, followed by resting at room temperature for 15–20 minutes. The reaction buffer containing 100 mM imidazole HCl, pH 6.6, 100 mM LiCl, 25 mM MgCl_2_, 2 mM EGTA, was added to the lipid micelles, in the presence of 0.2 μl of 1 M dithiothreitol, 5 μg of diglycerol kinase membranes, and dilution buffer (10 mM imidazole, pH 6.6, 1 mM diethylenetriaminepentaacetic acid, pH 7) to a final volume of 90 μl. The reaction was started by adding (γ^32^P) ATP solution and allowed to proceed at 25°C for 30 minutes. Lipids were extracted as described above and a 1.5 ml aliquot of the organic phase dried under nitrogen.

Lipids were then resuspended in methanol/chloroform (1/20) and 20 μl were spotted on a 20 cm silica gel thin layer chromatography plate. Plates were developed with chloroform: acetone: methanol: acetic acid: water (50:20:15:10:5), air dried, and subjected to autoradiography. The radioactive spots corresponding to ceramide-phosphate, the phosphorylated product of ceramide were scraped into a scintillation vial and counted on a scintillation counter. Linear curves of phosphorylation were produced over a concentration range of 0–640 pM of external standards (dioleoyl glycerol and C-III ceramide, Sigma). DAG and ceramide levels were normalized to lipid phosphate.

#### C. Lipid phosphate measurement

Lipid phosphates were measured according to the method of Rouser et al [20]. Briefly, 20% of the lipid sample was dried under nitrogen and oxidized with 70% perchloric acid on a heating block at 160°C for 45 minutes. The tubes were allowed to cool then water was added, followed by 2.5% ammonium molybdate, and 10% ascorbic acid with vortexing after each addition. The tubes were then incubated at 50°C for 15 minutes, allowed to cool, and absorbency read at 820 nm and compared to standards.

##### 3. Sphingomyelinase assay

Specimen from lung tissue stored in Sucrose-HEPES-Tris Cl (SHT) buffer was subjected to homogenization with 22G1/2 syringe followed with 27 G1/2 needle.

The activity of Acid SMase (A-SMase) was determined using a mixed micelle assay system. 10 μg of protein was mixed with a reaction mixture containing 10 nmol of [^14^C]Sphingomyelin (100,000 dpm) resuspended in 100 mM Sodium acetate, pH 4.5 and 0.1% Triton X-100 in a total volume of 100 μl. The reaction was incubated for 30 minutes at 37°C in shaking water bath and then was terminated by the addition of 200 μl of H_2_O. After phase separation, 300 μl of the upper phase containing the enzymatically released N-[^14^CH_3_]phosphocholine was counted in a liquid scintillation counter.

##### 4. Ceramidase assay

Specimen from lung tissue stored in Sucrose-HEPES-Tris Cl (SHT) buffer was subjected to fractionation in 25 mM and 100 mM Tris-Cl (pH 7.4), 100 mM Tris-CL (pH 9) and 0.5 M NaAc (pH 4.5). The collected membranes were then sonicated and subjected to the ceramidase assay.

Ceramidase activity was determined in an assay mixture of NBD C_12_-ceramide and Triton X-100 at final concentration of 100 μM and 0.3% respectively, in 0.5 M sodium acetate buffer, pH 4.5 (for acidic enzyme), 100 mM Tris-Cl buffer, pH 7.4 (for the neutral activity) and 100 mM Tris-Cl buffer, pH 9 (for the alkaline enzyme). The reaction was carried using 100 μg of protein in 50 μl of the specified buffer and mixed with 50 μl of the substrate. Samples were then incubated at 37°C in shaking water bath for 1 hour. The reaction was terminated by the addition of 300 μl chloroform/methanol (1:1, v/v) followed by vortexing and centrifugation at 2500 g for 10 minutes. 150 μl of the lower phase was transferred to a new microcentrifuge tube and dried. Samples were then resuspended in 40 μl chloroform and 35 μl was spotted on TLC and ran in chloroform/methanol/NH_4_OH (6.4 N) (90:30:0.5, v/v/v) for 1 hour. After air drying the plates were scanned on phosphoimager and the observed bands were quantified.

### 5. Bcl-2 and Bax expression

Tissue sections from normal and hyperoxic animals were incubated in 10 mM sodium citrate buffer, pH 6.0 at 95°C for 5 min and repeated two times. Slides were then allowed to cool in the buffer for 20 min and then washed in deionized water for two minutes and repeated three times. After the procedure of antigen unmasking is through, specimens were incubated in 1.5% goat serum followed by anti-Bcl-2 peptide antiserum or anti-Bax peptide antibodies (Santa Cruz Biotechnology). Slides were counterstained with propidium iodide. Bcl-2 and Bax immunostains were scored from 0 to 4, whereby 0 indicates 0% Bcl-2 and Bax positive cells, 1 indicates 1–25%, 2 indicates 25–50%, 3 indicates 50–75%, and 4 indicates 75–100%. Mod K cells were already reported to express Bax and were used as positive control for Bax immunostains and were scored 4. MCF 7 cells transfected with Bcl-2 were used as positive controls because of overexpression of Bcl-2 immunostains. MCF 7 cells were also scored 4 as well [21,22]. Pictures from endothelial, bronchial and alveolar areas were obtained and scored individually. The average score was then calculated.

### 6. E-Cadherin immunostaining and Hoechst staining

Antigen retrieval for tissue sections from control and hyperoxic animals was performed as described before for Bax and Bcl-2 immunostaining. The specimens were then incubated for 1 hr in 0.2% Triton-X-100 (for permeabilization) diluted in 3% normal goat serum, followed by 2 hrs incubation in E-cadherin antibody (Chemicon International, USA) diluted 1:500 in 1.5% normal goat serum. Thenafter, slides were incubated for 1 hr in FITC-conjucated secondary antibody. After several washes, tissues were counterstained with Hoechst staining. Hoechst (10 mg/ml) was diluted to 1 μg/ml, in which slides were incubated for 5 minutes, and then washed in phosphate buffered saline (PBS) for 45 minutes.

### 7. IL-1β expression

Total RNA was extracted from lung tissues preserved in liquid nitrogen according to the manufacturer using the MN Nucleospin^® ^RNA II extraction kit (Macherey-Nagel GmbH Co.KG, Düren, Germany). Reverse-Transcriptase PCR (**RT-PCR**) from the RNA template (1 μg) was performed according to the manufacturer using the Reverse-iT™ One-Step RT-PCR Kit (ABgene, Surrey, UK). Specific primers were used to detect the IL-1β as the β-actin gene for normalization purposes (figure 6A).

### Statistical assessments

Differences were considered significant for p < 0.05 in comparing two measurements. Comparison among groups was evaluated by analysis of variance (ANOVA). The Kruskall-Wallis test was used to compare apoptotic index scores.

## Results

**Figure 1:**
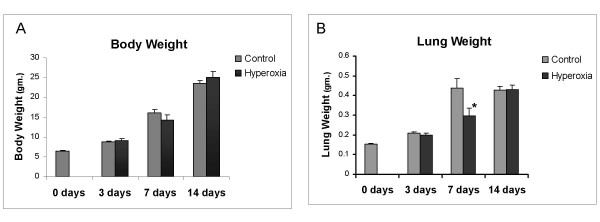
Body and lung weight changes of animals exposed to hyperoxia vs controls. * is placed where the difference is statistically significant. A) Body weights of the control vs. the hyperoxia animal group at 0, 3, 7 and 14 days. B) Lung weights of the control vs. the hyperoxia animal group at 0, 3, 7 and 14 days.

All rats survived the 7 days of hyperoxia. 50% mortality was observed by day 14 in the subgroup of animals that were exposed to 7 days of hyperoxia and were then placed in room air for additional 7 days. In addition, the pups that survived the 14 days appeared ill and weak. There was no statistically significant difference in body weights of the different animal groups when compared to controls (figure 1A). Lung weights showed no difference between the different control and hyperoxia group except for day 7 (Figure 1B); the hyperoxia-exposed group at day 7 weighed less than the control group.

**Figure 2:**
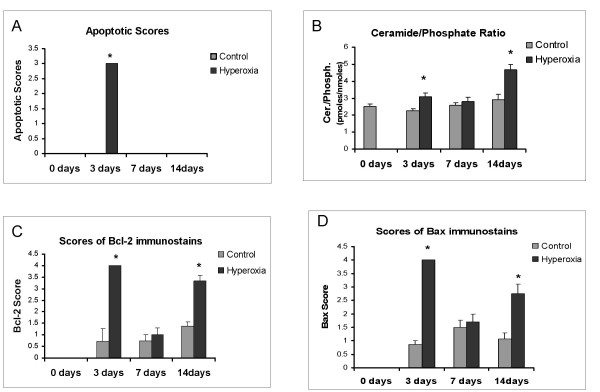
Changes in apoptosis, ceramide, Bcl-2 and Bax  of neonatal lungs exposed to hyperoxia. * is placed where the difference is statistically significant. A) Apoptotic scores in the whole lung of the control vs. the hyperoxia animal group at 0, 3, 7 and 14 days. B) Ceramide/ phosphpate ratio levels of the control vs. the hyperoxia animal group at 0, 3, 7 and 14 days. C) Scores of immunostains in the whole lung of the control vs. the hyperoxia animal group at 0, 3, 7 and 14 days. D) Scores of immunostains in the whole lung of the control vs. the hyperoxia animal group at 0, 3, 7 and 14 days.

To determine the role of the sphingolipid ceramide in hyperoxia, ceramide levels in lung tissue were measured and found to be 3.10 pmole/nmole phosphate (pmole/nmnmole Pi) and 2.26 pmole/nmole Pi on day 3, 2.82 pm/nmPi and 2.58 pm/nmPi on day 7 and 4.66 pm/nmPi and 2.91 pm/nmPi on day 14 in hyperoxia-exposed rats and control rats, respectively. The differences on days 3 and 14 were statistically significant (p < 0.01) (Figure 2A).

The pathways of ceramide up-regulation include *de novo *synthesis, sphingomyelinase activation, or inhibition of ceramide catabolism. Investigation of *de novo *synthesis of ceramide in an animal model is technically difficult because the most acceptable assay is the demonstration of radioactively-labeled palmitate incorporation into ceramide that is newly synthesized from the condensation of serine and palmitate. The amounts of radioactive palmitate that would be required are too large for the conduct of a safe experiment in this model. Also, measurement of ceramide synthase, the enzyme that is most important in *de novo *ceramide synthesis is exceedingly difficult in the setting of homogenized animal tissue. However, we were able to measure sphingomyelinase activity and found no change in the enzyme's activity in response to hyperoxia. Ceramidase(s) acidic, neutral or alkaline activity was also measured and also did not show any increased activity at the different time points when compared to the control groups. These findings suggest that neither sphingomyelinases nor ceramidases are involved in ceramide generation in the setting of hyperoxia. This raises the possibility that *de novo *synthesis is likely to be the mechanism by which ceramide accumulates in our model.

The role of Bcl-2 family of proteins was evaluated by the measurement of the anti-apoptotic protein Bcl-2 and the pro-apoptotic protein Bax. As explained in Methods, the expression of Bcl-2 was scored and was found to be 4 and 0.71 at day 3, 1 and 0.735 at day 7 and 3.33 and 1.38 at day 14 in the hyperoxia-exposed rats and the control rats, respectively The differences at days 3 and 14 were statistically significant (Figure 2B). As for Bax, the expression was 4 and 0.86 on day 3, 1.71 and 1.50 on day 7 and 2.75 and 1.07 on day 14 in the hyperoxia-exposed and control rats, respectively. The difference at day 3 and 14 was statistically significant (Figure 2C).

**Figure 3:**
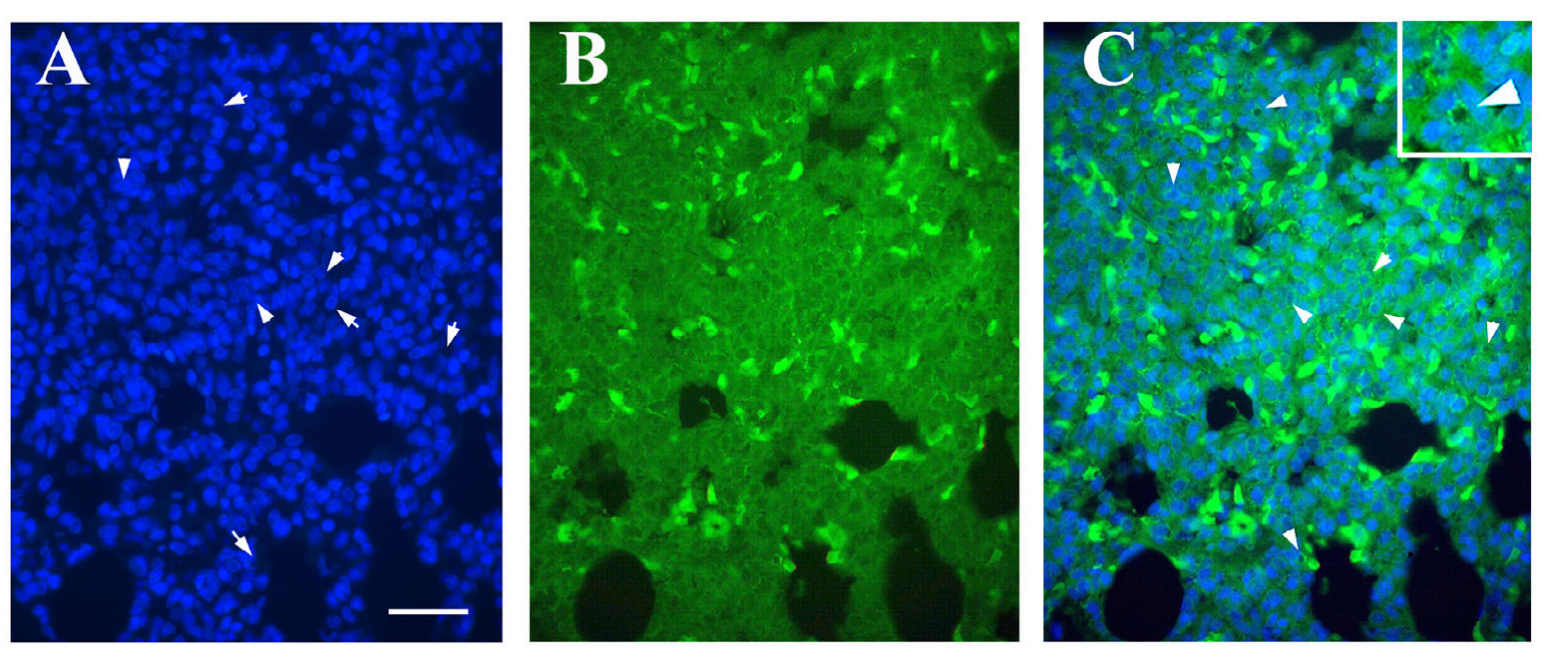
E-Cadherin and Hoechst Staining of neonatal lung after 3 days of Hyperoxia. A, B, and C correspond to the same section of the examined tissue. Bar corresponds to 40 μm. A. Hoechst stained cells are noted and the white arrows are pointing at cells with apoptotic nuclei. B. E-Cadherin stained cells. C. An overlay of Hoechst and E-Cadherin, arrows indicate the dense apoptotic nuclei surrounded by E-cadherin (halo) staining.

TUNEL staining was used to determine the level of apoptosis in experimental and control rats and to generate an apoptotic index, which was 0.65 for the lungs of hyperoxia-exposed rats at 3 days. The difference at 3 days was statistically significant (p < 0.001). Hoechst staining provided further evidence that apoptosis did occur at 3 days. The observation of relatively small and densely Hoechst-stained nuclei suggests that cells are apoptotic. To determine the histolology of cells undergoing apoptosis, E-cadherin immunostaining was performed. Cadherins constitute a family of transmembrane glycoproteins that are involved in Ca^2+^-dependent cell-cell interactions. They function in the maintenance of tissue integrity and morphogenesis [23]. E-cadherin is the predominant subclass that has been identified in most types of epithelial cells including lung epithelial cells, where it is closely associated with the adhesion belts of the adhering junctional complexes [24]. Hoechst staining, on the other hand, is utilized to document apoptosis by visualizing chromatin condensation and nuclear shrinkage followed by the formation of apoptotic bodies. By performing both (E-cadherin and Hoechst) stains simultaneously, we were able to demonstrate: a) that apoptosis occurred predominantly at 3 days and b) the main cells undergoing apoptosis were epithelial cells as they stained positive by E-cadherin antibody. (figure 3B and 3C).

**Figure 4:**
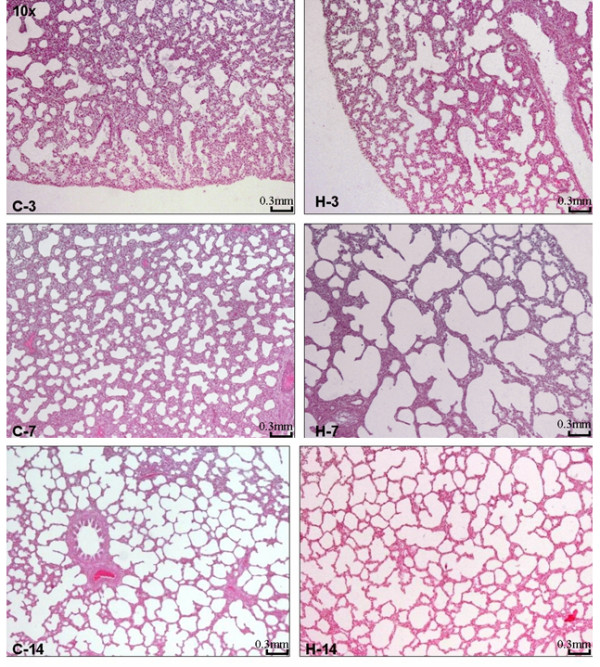
Histological findings of neonatal lungs in the presence of hyperoxia. Photomicrographs of lung tissue from control (C) and hyperoxia-exposed (H) rats at 3, 7, and 14 days. While normal development and alveolarization is seen in the control group, the hyperoxia exposed group shows significant dilatation and expansion of the alveolar ducts and the ventilatory units. The changes are most prominent at day 7 and regress at day 14. (Hematoxylin and Eosin × 10).

**Figure 5:**
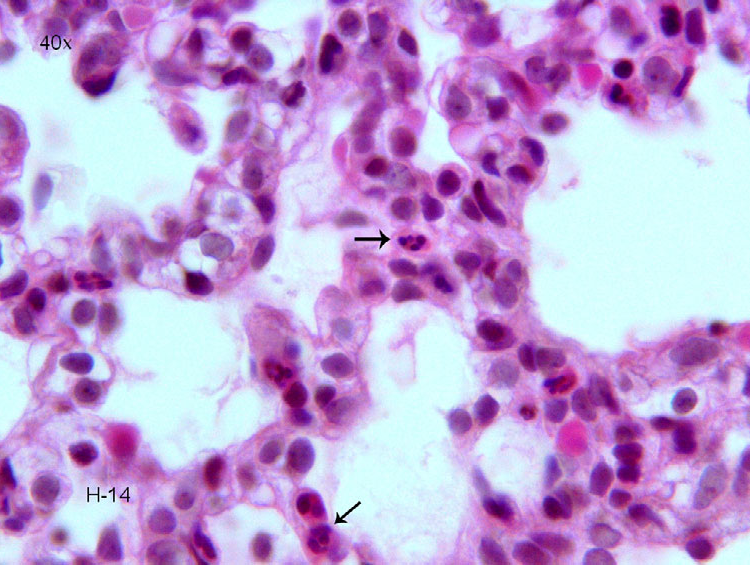
Acute lung injury after prolonged hyperoxia exposure. Photomicrograph of the interstitial wall of a hyperoxia-exposed rat at day 14 showing the prominent margination of neutrophils in the capillaries (Hematoxylin and Eosin × 40).

To evaluate the pathologic changes in response to hyperoxia, sections of lung tissue from control and hyperoxia-exposed rats were Hematoxylin and Eosin stained and examined by light microscopy. There were significant and striking differences. Most notably there was expansion and dilatation of the alveolar ducts and the ventilatory units including the air sacs. There was no apparent significant difference at day 3 but it became most exaggerated at day 7 (figure 4). The effect was sustained at day 14 but to a lesser degree. Over the course of the experiment, there was progressive thinning of the alveolar interstitial wall. At day 14, the density of the capillaries was the highest and in the hyperoxic-exposed rats, there was more marginating neutrophils and their number was increased when compared to the control group (Figure 5). There was no evidence of hemorrhage, hyaline membrane formation or coagulative necrosis.

**Figure 6:**
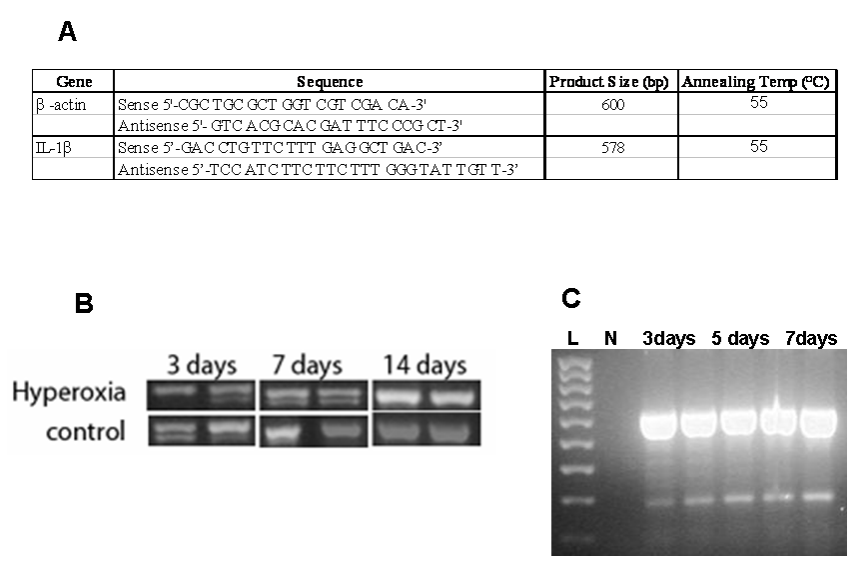
IL-1β expression in response to hyperoxia. A. Specific primers used to detect IL-1β gene and β-actin gene expression. B. Samples of RT-PCR amplicons corresponding to the IL-1*β *gene (578 bp) showing increased expression of IL-1*β *gene at day 14. C. Samples of RT-PCR amplicons corresponding to the *β-actin *gene. Lane L: 100 bp ladder, lane N: negative control, lanes 1–5: *β-actin *amplicons from samples at different dates.

The role of the inflammatory mediators in our model of lung injury was evaluated by measurement of IL-1β expression via Reverse-Transcriptase PCR (RT-PCR) method. β-actin gene expression was also evaluated as a control. There was an observable increase of band intensity for the IL-1 *β *gene at day 14 suggesting an increase in the inflammatory mediators expression in the hyperoxia groups compared to the control groups (Figure 6B).

## Discussion

Ceramide generation is an important step in the signaling cascades that lead to apoptosis. Ceramide is a sphingomyelin breakdown product that has not been studied in hyperoxia-induced lung injury. In this study, we examined the interplay of ceramide, Bax and Bcl-2 in a well-established model of hyperoxia-induced lung injury in the newborn rat [25,26]. Our study demonstrated overexpression of ceramide in neonatal lungs after 3 days of exposure to hyperoxia. Ceramide generation is usually by: a) The hydrolysis of membrane sphingomeylin via the activity of sphingomyelinases, b) The inhibition of ceramidases which hydrolyze ceramide to sphingosine and fatty acids, or c) by activation or *de novo *synthesis [11,13]. In our experiments, acidic sphingomyelinase did not change in response to hyperoxia at the three time-points examined, i.e. 3, 7, and 14 days. Also, ceramidase(s) activity did not change as well. These findings suggest that de novo synthesis is the likely mechanism by which ceramide accumulated in our animal model. Further testing of *de novo *synthesis for ceramide generation is technically difficult in an animal model. The significantly high levels of ceramide correlated with an increase in apoptosis as determined by TUNEL assay and an overexpression of Bax in hyperoxia-exposed lungs. The corresponding sections of the neonatal lungs after three days of hyperoxia did not demonstrate any evidence of cellular necrosis or loss of architecture of the lung parenchyma (Figure 3). This suggests that after 3 days of hyperoxia, cellular death occurs via apoptosis. Previous studies have shown similar results in the first 48–72 hours of hyperoxia. Kazzaz et al noted that in contrast to direct exposure of cells in culture to hyperoxia, apoptosis was clearly induced in the lungs of hyperoxic mice after 48 hours of hyperoxia [27]. Similarly, McGrath-Morrow et al demonstrated, after 3.5 days of high concentrations of oxygen, an increase in apoptosis in the peripheral lung of neonatal mice [28]. The increase in apoptosis was associated with an induction of Bax mRNA expression as well.

Bax appears to be a key activator of ceramide-mediated death pathways. Von Haefen et al demonstrated that ceramide-induced apoptosis is marginal in Bax-negative DU 145 cells. Reconstitution of Bax restored ceramide-induced apoptosis [29]. Buccellato et al. demonstrated that hyperoxia resulted in Bax activation at the mitochondrial membrane and subsequent cytochrome c release [30]. Pagano et al observed a progressive release of cytochrome c from the mitochondria into the cytosol of alveolar cells, following hyperoxic exposure. This release was accompanied by the translocation of the proapoptotic protein Bax [31]. The mechanism of the response of the Bcl-2 family of proteins to hyperoxia is thought to start with the release of cytochrome c from the intermembrane space into the cytosol accompanied by depolarization of the mitochondrial membrane potential. The release of cytochrome c leads to caspase 9 activation and the commitment of the cell to die. Apoptotic stimuli cause translocation of Bax from the cytosol to the mitochondria, where it causes membrane permeabilization. Anti-apoptotic proteins (Bcl-2, Bcl-x) prevent changes in the mitochondrial membrane potential. Antiapoptotic members of the Bcl-2 family inhibit membrane permeabilization by sequestering or inhibiting Bax and Bak. Interestingly, both ceramide and Bcl-2 family members appear to target the mitochondria.

At 7 days of age and after continuous exposure to hyperoxia, ceramide returned to baseline levels. This was accompanied by reduction in Bax and Bcl-2 expression to baseline levels and absence of apoptotic changes. The lack of apoptosis may represent adaptation of the animals to hyperoxia and/or it may represent a quiescence phase where the animals have managed to contain the initial hyperoxic injury. However morphologically, the lung tissue showed exaggerated expansion and dilation of the alveolar ducts and ventilatory units. Early margination of neutrophils within the capillaries of the interstitial wall is also noted. Jacqueline et al reported similar findings and concluded that hyperoxia disrupts the coordinated proliferation of the lung of neonates and, causes apoptosis of specific cell types that are needed in the development of the lungs of animals [32]. It appears that hyperoxia exposure and apoptosis observed at day 3 may have contributed to the delay in lung maturation and produced structural defects in the neonatal lungs of the hyperoxia group as well [33].

At 14 days, one week after discontinuing hyperoxia, there was another rise in ceramide without associated apopototic changes. A rise in Bcl-2 and Bax was also noted along with a change in the Bcl-2/bax ratio to favor Bcl-2. The lack of apoptosis at this point may result from the interplay of ceramide, a proapoptotic modulator, with Bcl-2, known to have antiapoptotic activity. The relationship between Bax and antiapoptotic proteins of the Bcl-2 family may also determine apoptosis [34-36]. Sawda et al, for example, demonstrated that the exposure of C6 glioma cells to etoposide resulted in apoptosis, increased formation of ceramide and a decreased expression of Bcl-2 with reciprocal increase in Bax protein. Reducing ceramide formation, prevented apoptosis and resulted in a change of the Bax/Bcl-2 ratio to favor Bcl-2 [37]. This indicates that ceramide's apoptotic effects maybe partially through its effects on Bax/Bcl-2 ratio.

The interaction between the Bcl-2 family of proteins and ceramide can be upstream or down stream. Without blocking ceramide accumulation, Bcl-2 has been shown to block ceramide-induced apoptosis. Vincristine, for example, increases ceramide levels in the presence of overexpressed Bcl-2 without apoptosis. This indicates that Bcl-2 anti-apoptotic effects act downstream of ceramide. The interaction of ceramide and Bcl-2, however, appears to be more complex: In the same cell type, Molt-4 cells, ceramide accumulation is induced by γ-irradiation, but this accumulation is prevented by Bcl-2 overexpression, indicating that Bcl-2 may function upstream as well [16].

In our study, there was an increase in Bax occurring at 3 days of age. This increase correlated with the apoptotic changes documented by TUNEL. Bcl-2 was elevated at 3 and 14 days. The Bcl-2 to Bax ratio (1, 0.58 and 1.21 at days 3, 7 and 14 respectively) did not appear to correlate with the apoptotic changes.

The rise of ceramide, Bax, and Bcl-2 may also be secondary to inflammation and lung injury. At 14 days, one week after discontinuing hyperoxia exposure, 50% mortality was noted and the animals that survived the experiment appeared very ill. IL-1β activity was also significantly increased at 14 days. It is evident that clear and irreversible changes had occurred and inflammation leading to the animals death. Lung tissue showed persistence of the expansion and the dilatation of the alveolar spaces but to a lesser degree when compared to day 7. However, there was noticeable increase in the number of marginating neutrophils in the capillaries of the interstitial wall when compared to the day 7 group (Figure 5).

Those findings suggest that prolonged hyperoxia in newborns can cause delayed lung damage characterized earlier by margination of neutrophils and later by interstitial and intra-alveolar edema. Hyperoxia may have also triggered proinflammatory cytokines and a cascade of events that lead to influx of neutrophils and irreversible lung injury. This has occurred even after placing the animals in room air for more than a week.

In conclusion, our study suggests that the effects of hyperoxia on the lung are time sensitive processes that include initial and delayed response. In our study, the initial response is characterized by a rise in ceramide, Bax and Bcl-2 leading to apoptosis. No evidence of inflammatory process is noted at this stage. In the delayed phase, no apoptosis was detected and the late phase is acute lung injury with influx of neutrophils and inflammatory response. No apoptosis is noted and the rise in Ceramide, Bax and Bcl-2 may be due to the inflammatory process. Apoptosis noted in the initial phase may then be an appropriate response through which damaged cells are removed without triggering an acute inflammatory reaction. As apoptosis is switched off, inflammatory process is activated leading to acute lung injury and death. We recommend further time points and experiments to delineate further the effects of hyperoxia. This will help us in planning interventions in a timely manner to counter the effects of hyperoxia and acute lung injury.
